# Challenges and opportunities in analyzing and modeling peptide presentation by HLA-II proteins

**DOI:** 10.3389/fimmu.2023.1107266

**Published:** 2023-03-29

**Authors:** Hesham ElAbd, Petra Bacher, Andreas Tholey, Tobias L. Lenz, Andre Franke

**Affiliations:** ^1^ Institute of Clinical Molecular Biology, University of Kiel, Kiel, Germany; ^2^ Institute of Immunology, University of Kiel, Kiel, Germany; ^3^ Proteomics & Bioanalytics, Institute for Experimental Medicine, University of Kiel, Kiel, Germany; ^4^ Research Unit for Evolutionary Immunogenomics, Department of Biology, University of Hamburg, Hamburg, Germany

**Keywords:** HLA-II & autoimmunity, immunogenicitiy, TCR - T cell receptor, mass-spectrometry (MS) based immunopeptidomics, neoantigen, immunotherapy

## Abstract

The human leukocyte antigen (HLA) proteins are an indispensable component of adaptive immunity because of their role in presenting self and foreign peptides to T cells. Further, many complex diseases are associated with genetic variation in the HLA region, implying an important role for specific HLA-presented peptides in the etiology of these diseases. Identifying the specific set of peptides presented by an individual’s HLA proteins *in vivo*, as a whole being referred to as the immunopeptidome, has therefore gathered increasing attention for different reasons. For example, identifying neoepitopes for cancer immunotherapy, vaccine development against infectious pathogens, or elucidating the role of HLA in autoimmunity. Despite the tremendous progress made during the last decade in these areas, several questions remain unanswered. In this perspective, we highlight five remaining key challenges in the analysis of peptide presentation and T cell immunogenicity and discuss potential solutions to these problems. We believe that addressing these questions would not only improve our understanding of disease etiology but will also have a direct translational impact in terms of engineering better vaccines and in developing more potent immunotherapies.

## Introduction

The human leukocyte antigen class II (HLA-II) proteins are a class of glycoproteins that are mainly expressed on the surface of antigen presenting cells (APCs) where they present peptides to CD4^+^ T cells. These peptides are derived from within the APCs itself, *e.g.* through autophagy (1), or from the cellular microenvironment, *e.g.* through receptor-mediated endocytosis (2) and macropinocytosis (3). As a result, APCs are able to present self-peptides together with foreign peptides, *e.g.* pathogens-driven peptides, to T cells. Thus, by identifying and analyzing the set of peptides presented on the surface of APCs, the set of potential T cell epitopes can be narrowed down considerably into a manageable set of peptides and proteins. Nonetheless, HLA-II genes are extremely polymorphic, with most of the observed genetic variants located inside the peptide binding pocket, indicating that different HLA-II alleles encode for proteins that present different peptides, *i.e.* different alleles result in different immunopeptidomes. Moreover, HLA-II proteins present a diverse set of peptides, hence, the combination of these two factors results in an astronomically large number of peptide HLA-II complexes. Consequently, a lot of effort has been directed toward identifying and characterizing the set of HLA-II presented peptides for a plethora of aims, ranging from investigating autoimmune diseases (4) to neoepitope identification and cancer immunotherapy (5–7).

The rise of MS-based immunopeptidomics during the last decade has revolutionized our ability to identify peptides presented by HLA proteins *in vivo.* This has led to a “*Cambrian explosion*” in the field of neoepitope identification where continuous efforts are being directed at improving the experimental (8) and the computational aspects (9–11) of the technology to enable an accurate identification of neoepitopes. For example, Feola et al. (12) have recently described an immunopeptidomics-based pipeline for neoantigen identification and cancer vaccine development. Meanwhile, Chong and colleagues have recently developed *NewAnce* for identifying non-canonical peptides presented by HLA proteins on tumors (13). Beside identifying presented peptides and neoepitopes, a lot of efforts has been directed at improving the quantification of peptides presented by HLA proteins (14, 15).

MS-immunopeptidomics has been also utilized for vaccine candidate prioritization. Recently, Bettencourt et al. (16) utilized this technology for identifying *Mycobacterium tuberculosis* (Mtb) presented peptides, subsequently, a proof-of-concept vaccine against Mtb was developed using a subset of the presented peptides. Further, an mRNA-based vaccine against *Listeria monocytogenes* was recently developed by Mayer and colleagues (17) using an MS-immunopeptidomics guided approach. MS-immunopeptidomics was also utilized for mapping the presentation of SARS-CoV-2 spike glycoprotein proteins by common HLA-DR proteins (18).

Despite the recent progress, deciphering the principles governing peptide generation from potential protein candidates has proven to be an extremely challenging task. A reliable prediction of immunogenicity and immunodominance is therefore still lacking. In addition, the impact of the cellular microenvironment, genetic alterations in the non-HLA components of the peptide presentation machinery, the ageing of the host cells and other environmental factors on peptide presentation by HLA-II proteins is still far from being completely understood. In this perspective, we thus highlight five open questions that are likely to improve our understanding of the biology of HLA-II peptide presentation and its influence on T cell immunogenicity.

## What is the role of digestive enzymes in shaping HLA-II immunopeptidomes?

Arguably, one of the crucial and, nonetheless, not-completely understood steps in peptide presentation by HLA-II proteins is the loading of HLA-II molecules. Conceptually, loading can be divided into two processes that operate asynchronously. Firstly, peptide generation, and secondly, the actual molecular loading. The former happens through the sequential cleavage of proteins inside the lysosome (19–21) which can be derived from, for example, self-proteins (22, 23), from microbes (16) or from environmental sources such as pollen allergens (24). Proteins in the lysosomes are mainly cleaved using a group of proteases referred to as cathepsins (Cat) (25). Although some cathepsins are ubiquitously expressed in all tissues, *e.g.* Cat B (26), Cat D (27), and Cat L (28), some show a tissue-restricted expression, for example, Cat S (28) in APCs.

Recent findings have suggested an important role for cathepsins in shaping peptide presentation and immunogenicity. For example, Riese et al. (29) identified a critical role for Cat S in antigen presentation in B cells and dendritic cells. Meanwhile, Dheilly et al. (30) have shown that the same enzyme also regulates antigen presentation by non-Hodgkin Lymphoma. Besides Cat S, Cat L has been shown to be a major regulator of presentation by epithelium cells (31) and in the production of an IgA antibodies against *M. pneumonia* (32). Cat L has also been shown to impact the specialization of the generated T cell response where treating *Leishmania major* infected mice with Cat L inhibitors worsen the disease by changing the T cell response from a Th1 to a Th2 response (33). Moreover, inhibiting Cat L in Non-obese diabetic (NOD) mice protected these mice form developing type I diabetes supposedly by increasing the fraction of T regulatory cells in these mice relative to untreated mice (34). These findings illustrate the need to study the influence of cathepsins on HLA-II peptide presentation in depth using recent technological advances in MS-immunopeptidomics and other omics fields. The provided insights would help in establishing a link between the digestive capacity of the cell (*i.e.* which digestive enzyme is expressed and at what level) and presentation by HLA-II proteins.

Different methods can be used to delineate the role of cathepsins in shaping HLA-II immunopeptidomes such as the combination of mass spectrometry (MS)-based immunopeptidomics and gene-editing methods. This can be accomplished either by knocking down one of the cathepsins in a cell culture and measure its impact on the immunopeptidome and/or by using a mixture of knock-ins and knockouts of different combinations of digestive enzymes and subsequently investigate the impact on the immunopeptidome. Machine-learning methods could then be set up to extract generalizable patterns from these experiments.

## What is the impact of cell type and cellular micro-environment on HLA-II presentation?

Although the experiments proposed above would provide much-needed insights into the role of proteases in shaping HLA-II immunopeptidomes, it is only one piece of the larger puzzle as it only delineates the determinants of the digestive process. Nonetheless, the actual pool of proteins available to these proteases*, i.e.* the pool of substrate proteins, is also still poorly understood. Recently, Wang et al. (4) have described differences in peptides presented by B cells and monocytes, and argued that differences in the preprocessing machinery and the available proteins to each cell-type might explain this difference. Along these lines, Marcu et al. (35) have revealed qualitative and quantitative differences in the immunopeptidome of different tissues. Further, presentation of viral and tumors proteins has been shown to increase after tagging these proteins for autophagy (36–39), pointing to an important rule for autophagy and the non-HLA components of the presentation machinery in modeling the set of presented peptides. These components can also be influenced by external and internal signals such as inflammation, stress, and calorie restriction (40–42). Therefore, future efforts should focus on deciphering the link among the cellular status, the cellular micro-environment and HLA-II immunopeptidomes, for example, using simultaneous deep multi-omics profiling, *e.g.* proteomics and transcriptomics, coupled with MS-based immunopeptidomics across different cellular states and across different tissues and organs.

## What are the temporal aspects of HLA-II presentation?

An additional challenge is the extent of variation in the duration of presentation, *i.e.* the duration a given peptide is presented on the cell surface by an HLA-II protein. Differences in the lifespan, *i.e.* duration of presentation, of different peptide HLA-I complexes have been proposed to be a major factor shaping the immunogenicity of different peptides (43, 44). For example, Micheletti et al. (45) have identified an important rule for the lifespan of HLA-I peptide complexes and the efficiency of the generated T cell response. Despite the prominent role of presentation dynamics in shaping immunogenicity, it is a poorly understood and characterized process.

Different experimental approaches can be utilized to investigate this problem, for example, by recording the presentation dynamic of a knocked-in gene-of-interest, *e.g.*, specific antigenic proteins or tumor-associated neoantigen with a controllable level of expression. In such systems the link between expression levels and temporal presentation behavior can be delineated by varying either the strength and/or the duration of expression and quantifying the effect on presentation dynamics using immunopeptidomics.

## How do post-translational modifications shape HLA-II immunopeptidome formation and how is this linked to the cellular state?

As illustrated above, the immunopeptidome is an extremely complex, dynamic, and only partially understood entity. One of the factors that require further investigation are post-translational modifications (PTMs), for example phosphorylation, acylation or deamination. Such PTM can change the physicochemical interaction between peptides and HLA-proteins (46) as well as the interaction between a peptide-HLA complex and TCRs and, as a result, affect the generated T cell response (47). Indeed, in different autoimmune diseases such as type I diabetes some of the target antigens were found to harbor PTMs, for example, insulin/proinsulin (48) and GRP7 (49).

Multiple challenges render the identification of PTMs in the immunopeptidome troublesome. For example, peptides with PTMs have a low abundance or at least a lower abundance relative to the un-modified peptides (50) (at least this holds true in proteomics). Moreover, some PTMs might interfere with peptide ionization and hence decrease the detectability by MS (50). Besides these experimental challenges, there are computational challenges associated with identifying PTMs. Mainly, the database search which becomes computationally impractical when a large number of PTMs is included (51). Nonetheless, through the development of PTM-aware search engines such as *MSFragger* (52)*, and MODa *(53), or through the developed of tailored pipelines such as PROMISE (54) progress is being made in addressing this challenging task.

## What are the factors that shape the T cell response to a particular peptide-HLA complex?

**Figure 1:**
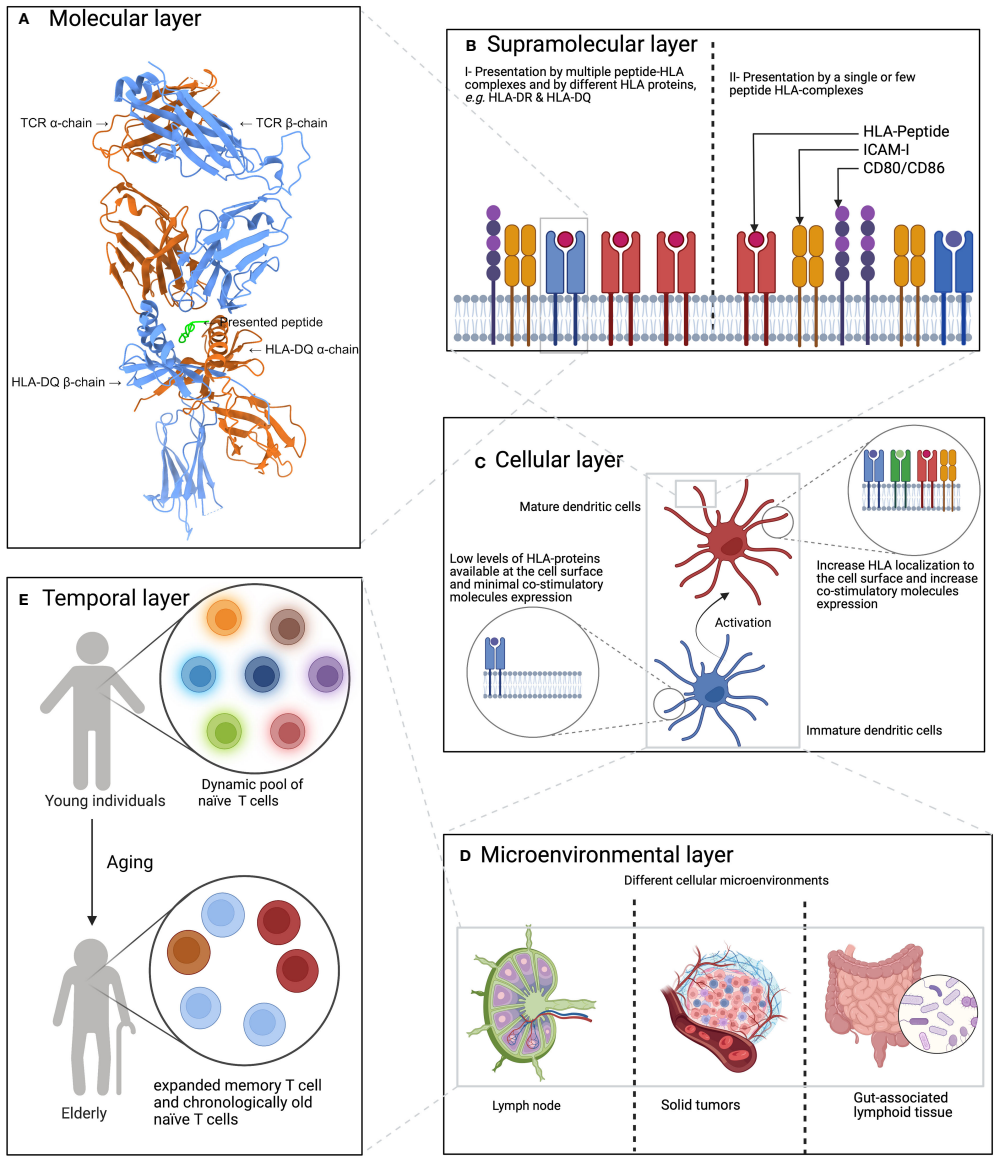
A hierarchal model of factors influencing or shaping the T cell response. **(A)** The molecular layer which focuses on HLA-peptide-TCR interaction. Here, the interaction between HLA- DQ 2.2 loaded with a gluten-derived peptide and a TCR is shown. The panel was created using ChimeraX (55), the protein 3D structure was obtained from Protein Data Bank (56) under the identifier 6PX6 (57). **(B)** The supramolecular layer which describes the impact of co-stimulatory molecules and the count of HLA-P complexes, among others. **(C)** Describes the impact of the cell types on both sides of the immunological synapse,*i.e.* the type of APCs and T cells, on shaping the generated T cell response. **(D)** The CME layer which describes the impact of the cellular micro-environment on shaping T cell response where extra cellular signaling can influence both antigen presentation, T cell priming and subsequently the generated T cell response. **(E)** The impact of aging on shaping T cell response where the T cell pool becomes dominated by expanded memory T cells and chronological old naïve T cells.

Conceptually, factors shaping the interaction between an APC and a T cell can be broken-down into five interwoven layers (**Figure 1**): starting with (A) the molecular layer, then (B) the supramolecular layer, followed by (C) the cellular layer, then (D) the micro-environmental layer, and finally (E) the temporal layer (see below).

### Molecular layer

The molecular layer refers to the interaction between an HLA-peptide complex and a TCR. At this level, the sequence-based physicochemical properties of the three molecular components would determine which set of TCRs will recognize a particular peptide-HLA complex. The strength of this interaction also governs different aspects of the generated T cell response, for example, Tubo et al. (58) have recently shown that the strength of TCR-peptide-MHCII interaction have an important role in shaping the differentiation of naïve CD4^+^ T cells.

### Supramolecular layer

The supramolecular layer involves the formation of an immunological synapse (59) between an APC and a T cell where a collection of co-stimulatory molecules and their receptors, along with the TCR and its intracellular signaling components, assemble to form an efficient signaling and communication channel between the two cells. One of the open questions here is the impact of presentation density on T cell activation, *i.e.* the absolute number of HLA-II proteins presenting the same peptide, and which HLA-II proteins present the peptide to the T cell. Previous work by Irvine et al. (60) illustrated the exceptional sensitivity of CD4^+^ T cells towards MHC-II molecules where a single MHC-peptide complex is sufficient for driving the T cell response. Hence, a potential future direction is to disentangle the impact of affinity, *i.e.* the affinity of a particular TCR toward a particular HLA-peptide complex, from the impact of the count, *i.e.* the number of HLA-peptide complexes.

### Cellular layer

The cellular level refers to the type of APCs and T cells: although HLA-II molecules are mainly expressed on professional APCs, their expression can be induced on a variety of other cell types by inflammatory cytokines, *e.g.* INF-γ (61, 62). Additionally, different cell types can act as professional APCs such as dendritic cells and macrophages (63) each of which is also subclassified further into different cell types. For example, dendritic cells are classified into conventional dendritic cells (cDCs) and plasmacytoid dendritic cells, inflammatory dendritic cells and Langerhans cells (64). cDCs are further subclassified into cDC1 and cDC2 (65), each with a different phenotype and function. For example, cDC1 are efficient cross-presenters (65, 66) while cDC2 cells can efficiently prime naïve CD4^+^ T cells (65, 67, 68). On the other side of the immunological synapse, T cells can exist in different states, for example, naïve, effector, memory, and anergic T cells, each of which have a different antigenic sensitivity (69, 70), and even utilize different metabolic pathways (71). Hence, characterizing the cellular impact on presentation and T cell activation is of paramount importance for a deeper understanding of immunogenicity.

### Microenvironmental layer

The cellular micro-environment (CME) can play a vital role in shaping either antigen presentation and/or the resulting T cell response. In the former, the CME plays a pivotal role in determining which proteins are available to the presentation machinery along with finetuning the cellular state, *e.g.* which digestive enzymes are expressed and/or which co-stimulatory molecules are expressed through cellular signaling (72–74). For the T cell response, CME signaling can have a crucial role in shaping the behavior of T cells, *e.g.* TGF-β and the induction of regulatory T cells (75).

### Temporal layer

Finally, the temporal level which refers to the (immune system) age of the host along with previous exposure to the same antigen or to a similar antigen. These two factors will likely shape the resulting response greatly by changing the pool of available T cells, *e.g.* in young individuals the T cell pool is shaped by more dynamic naïve T cells, while in older individuals it is mainly characterized by expanded memory T cells and chronologically older naïve T cells (76). Hence, a deeper characterization of the impact of aging on antigen-presentation and T cell expansion is vital because it might provide strategies to design vaccines that would work on the elderly, which has proven to be a challenging task, too (77, 78).

## Concluding remarks

Recent advances in computational (9, 10) and experimental approaches, *e.g.* improved sensitivity of MS analysis, are enabling much deeper and more accurate characterization of the immunopeptidome. Nonetheless, we are still far from a complete understanding of all the factors shaping the formation of the immunopeptidome and its dynamics. Furthermore, despite the recent improvement in predicting peptide-presentation *in vivo*, this improvement has not translated into a reliable *in silico* prediction of immunogenicity. Indeed, factors governing immunogenicity and immunodominance are still poorly understood. In this perspective we aimed at highlighting gaps in our understanding of peptide presentation and T cell activation, and discussed approaches for further investigation. Coordinated, systematic and large-scale efforts are urgently needed to provide deeper insights into antigen presentation and T cell activation. These insights would help developing more efficient vaccines, better immunotherapies, and also improve our understanding of immunity and host-pathogen interactions more generally.

## Data availability statement

The original contributions presented in the study are included in the article/supplementary material. Further inquiries can be directed to the corresponding author.

## Author contributions

HE and AF conceived the manuscript. HE wrote the manuscript with input from TL, AT, PB and AF. All authors read and approved the final manuscript.
